# Unveiling population-specific outcomes: Examining life cycle traits of different strains of *Chironomus riparius* exposed to microplastics and cadmium questions generality of ecotoxicological results

**DOI:** 10.1371/journal.pone.0304739

**Published:** 2024-07-10

**Authors:** Halina Binde Doria, Vivian Wagner, Quentin Foucault, Markus Pfenninger

**Affiliations:** 1 Department Molecular Ecology, Senckenberg Biodiversity and Climate Research Centre, Frankfurt am Main, Germany; 2 LOEWE Centre for Translational Biodiversity Genomics, Senckenberg Biodiversity and Climate Research Centre, Frankfurt am Main, Germany; 3 Institute for Molecular and Organismic Evolution, Johannes Gutenberg University, Mainz, Germany; 4 Department Evolutionary Genetics, Bielefeld University, Bielefeld, Germany; Universidade Regional Integrada do Alto Uruguai e das Missoes, BRAZIL

## Abstract

Ecotoxicological tests used for risk assessment of toxicants and its mixtures rely both on classical life-cycle endpoints and bioindicator organisms usually derived from long-term laboratory cultures. While these cultures are thought to be comparable among laboratories and more sensitive than field organisms, it is not well investigated whether this assumption is met. Therefore, we aimed to investigate differential life-cycle endpoints response of two different strains of *C. riparius*, one originally from Spain and the other from Germany, kept under the same laboratory conditions for more than five years. To highlight any possible differences, the two populations were challenged with exposure to cadmium (Cd), polyvinyl chloride (PVC) microplastics and a co-exposure with both. Our results showed that significant differences between the strains became evident with the co-exposure of Cd and PVC MPs. The German strain showed attenuation of the deleterious Cd effects with microplastic co-exposure in survival and developmental time. Contrary to that, the Spanish strain showed no interaction between the substances. In conclusion, the toxicity-effects of contaminants may vary strongly among laboratory populations, which makes a universal risk assessment evaluation challenging.

## 1. Introduction

The effects of microplastics (MP) alone or in complex mixtures with toxic pollutants like sewage discharges, nanoparticles, surfactants and metals [1–4] are currently under intense research in the ecotoxicological and risk assessment community [5–7].

Although several studies targeting MPs are focusing on new and innovative techniques to identify early signs of deleterious effects on organisms [8–10], validated and thus legislatively relevant OECD tests still heavily rely on classical life-cycle endpoints [11–13]. Therefore, life cycle bioassays play a crucial role for decision making process regarding risk assessment of toxic substances, and are in addition reliable and cost-effective tools [14,15], especially when combined with the measurement of a broader range of fitness parameters [13].

It is known that laboratory strains tend to be more sensitive than field organisms when exposed to environmental toxicants [16], thus probably leading to an overestimation of toxic effects on field organisms. While this is in line with the precautionary principle, the use of different laboratory strains is questioned for generating often conflicting and idiosyncratic results, thus impacting the generality of the assessment results [17,18]. These concerns appear valid, since laboratory populations are generally inbreed, therefore less genetically diverse and, most importantly, differentially diverged [18,19]. In addition, they may be differentially adapted to the particular laboratory environment they are maintained at [24]. Thus, they may tend to idiosyncratic, non-representative phenotypical reaction norms that can lead to can lead to highly diverse, non-replicable responses when challenged by the same toxicant in different laboratories.

*Chironomus riparius* is a validated freshwater species for toxicity tests [12,20]. Due to its abundance, it is basic to benthic food webs and transfers biomass from water to terrestrial environments, as larvae and as adults, respectively [21]. In natural environments its effective population size can reach ~10^6^ individuals [22], while laboratory strains usually have 200 to 1000 individuals as effective population size [23,24]. Thus, it is an interesting model to study differential strain responses to toxicants. Indeed, Nowak et al. [19,42] have investigated the genetic diversity of several *C. riparius* European stock cultures and their influence on ecotoxicological tests. Although they have concluded that laboratory strains were genetically impoverished due to inbreeding, which affected the results of the bioassays, the different evolutionary history of each population as well as the different keeping conditions were not in the focus of the study.

In the present study we aimed to investigate the differential response of two *C. riparius* strains with distinct evolutionary trajectories, kept under the same laboratory conditions for more than five years. One population originated in southern Spain, the other population was sampled in Germany [25]. Apart from their proven differential adaptation to the different climates (Waldvogel et al. 2018 [33]), their habitats of origin likely differed in many more aspects, which is reflected in many genomic regions that are more diverged than expected by genetic drift alone.

The two strains were challenged with the LOEC (Lowest Observed Effect Concentration/Level) concentration for Cd [11], an environmentally relevant concentration of PVC MPs [9] and a mixture of the two substances, which is said to have either synergetic [26] or antagonist [27,28] effects, depending on the organism evaluated. Polyvinyl chloride (PVC) MPs are among the most used polymer in civil construction e.g. as an alternative for sand in concrete mixtures [29]. Cadmium (Cd) is a non-essential and highly toxic metal [30,31]. Its presence is widespread due to Cd being present in fertilizers and manufacturing process of paints and plastics [30,32]. With this study design we aimed to answer (a) whether there is a potential modulation of chronic Cd toxicity by co-exposure with PVC MPs, and (b) whether there are possible differences of sensitivity to Cd, PVC and their mixture between the two distinct laboratory populations.

## 2. Materials and methods

### 2.1. Culturing conditions

The *C. riparius* populations used in the present study are maintained in the same laboratory as an in-house laboratory culture since 2016 until the start of the described experiments in 2022. The one originated in southern Spain (Andalusia; 37.399080°N, -4.5267980°E) (SP) was uniquely sampled in 2016 [25,33]. The population collected in Germany (DE) was sampled at different time points for three years (2016–2019) (Hasselbach, Hessen, Germany; 50.167562°N, 9.083542°E) [25,34,35]. Both populations were obtained from natural but anthropogenically remodelled streams. Apart from the obvious geographically and resulting climatically different conditions, these probably also differed in many other, selectively relevant aspects, such as water chemistry, water regime, emissions, etc‥

Both stocks are meticulously kept apart to avoid their mixing. Culturing conditions of both of the cultures follows a modified version of the method described in the OECD guideline N°219 and already published by Foucault et al. (2019) [36]. Briefly, the cultures are continuously aerated and maintained at constant temperature of 20 ºC, 60% of humidity and under a 16:8 photoperiod. Larvae are raised in large trays with 1:4 ratio of sediment:medium. Sediment consists in washed (pH neutral) playground sand. Medium is deionised water adjusted to a conductivity of 520–540 μS/cm with aquarium sea salt (e.g., TropicMarin®) and a basic pH around 8. The organisms were fed daily with 0.4 g finely grounded fish food (e.g., Tetramin® Flakes). This food was also used for all subsequent life cycle experiments.

### 2.2. Test compound and chemical analyses

Cadmium chloride salt (Merck, Germany) was used as Cd source. Stock solutions of 10mg/L were made in deionized dechlorinate water. Final nominal concentration of 50 μg.l-1, selected as LOEC [11], was reached by diluting 7.5 ml of the stock solution directly into the bowls on day 1 of the experiment. PVC powder with particle sizes from 0–200 μm were purchased from PyroPowders, Erfurt, Germany and sieved by vibratory sieve shaking (mesh pore size of 100 μm), to a final particle size of < 100 μm. This size range was selected to be within the particles feeding range for 3rd and 4th larval stage of *C. riparius* [37]. The microplastic powder was added to the dry sand at a 1 g.kg−1 concentration, which reflects a sublethal environmentally relevant scenario [38,39]. The Cd and PVC MPs co-exposure was achieved by combining both exposure scenarios on the concentrations above described. Cd actual toxicant concentrations in the overlaying waters and sediment can be found in [11]. No toxicants from PVC leachate were identified in the replicates exposed to MPs.

### 2.3. Life-cycle toxicity test

The chronic exposure test was carried out for one generation according to OECD guideline 233 [12] with slightly modifications. Test vessels were kept under the same conditions as the *C. riparius* stock cultures described above. Water evaporation in the test vessels was compensated for by adding demineralized water. Conductivity was maintained between 540–650 μS/cm and pH around 8. The experiment was design to consist in four different treatments: negative control, with neither Cd nor PVC, (CTRL); 50 μg. L-1 of Cd (Cd); 1 g.kg−1 of PVC microplastics (PVC); and the combination of the Cd and PVC treatments (Cd + PVC) for each experimental population: Spain (SP) and Germany (DE). Totalizing eight experimental groups each one containing five replicates for biological analysis.

Three days prior to the start of the experiment, 10 freshly laid egg ropes were separated from each stock culture into 6-well plates (3 mL of medium, for details see Foucault et al. 2019 [36], per well). Five fully hatched egg ropes from each experimental population were chosen to start the experiment. On the same day, 30 glass bowls (Ø20 × 10 cm) filled with a 1.5 cm sediment layer composed by washed, pH neutral, playground sand and 1.250 L of medium were prepared, and aeration started. Each bowl was initiated by adding 30 first instar larvae in each one and aeration was suspended for five hours to allow the animals to reach the sediment. The replicates were fed according to a feeding schedule (S1 File).

Emerged individuals and their sexes were recorded daily to calculate survival, sex ratio and median developmental time (EmT50), being the last parameter calculated according to Oppold et al. (2016) [25]. All adults from each replicate were then collected and placed together in a single reproduction cage according to the exposure scenario, resulting in eight different breeding cages. Egg ropes were recorded and collected daily and number of eggs in each clutch were counted to estimate fertility as described in Foucault et al. (2019) [36]. Finally, to monitor overall population fitness, estimations of the population growth rate (PGR), an integrated endpoint in the life-cycle experiment, was done also according to [40].

### 2.4. Data analysis

Data was tested for normality with Shapiro-Wilk test and homogeneity of variances with Levene’s test. Differences between populations and exposure scenarios were checked using one-way analysis of variances (ANOVA) followed by Tukey’s post hoc test. When not homogenous and normal distributed data was analysed with Kruskal-Wallis nonparametric test followed by Dunn’s post-hoc test. All statistical analysis was done with the software R (Version 4.0.0) using the packages *dplyr* for ANOVAs, and the package *car* for post hoc tests. Descriptive results can be found in the S1 and S2 Tables in S1 File.

Finally, a linear mixed model was used to investigate interaction between populations and treatments on survival, EmT50 and fertility in the present experimental conditions with the replicates per treatment (rep) as random factor followed by ANOVA test. The statistical analysis was performed using R (Version 4.0.0) using *lme4* package for linear mixed‐effects model and the package *car* and *PMCMR* for post-hoc test [41].

## 3. Results

### 3.1. Emergence, EmT50 and fertility

**Fig 1:**
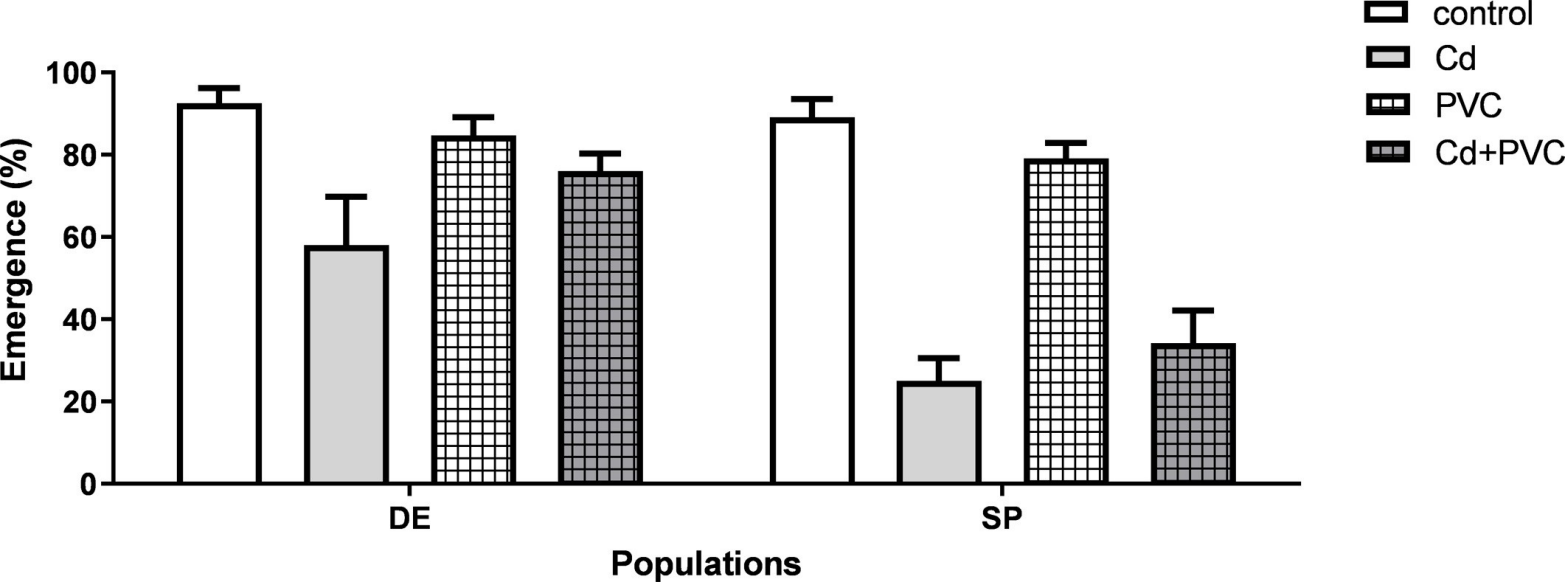
Survival represented by percentage of emerged adults in *C. riparius* of two different populations originated from Spain (SP) and Germany (DE) after exposure to 50 ug. L-1 of Cd (Cd), 1 g.kg-1 of PVC MPs (PVC), the mixture of the previous two exposure scenarios (Cd+PVC) and the control group. Data is expressed as mean values ± standard error. Different letters indicate significant differences between groups (p <0.05).

The total emergence of the control groups was 91.1±9.6% for the SP population and 92.5±7.4% for the DE population, which was well above the 70% survival by the OECD guideline for test validation (Fig 1). Statistical differences in survival between the treatments and in the two strains were observed when organisms were exposed to Cd in comparison to the control and PVC groups. The co-exposure of Cd and PVC exhibited conflicting results between the populations. The DE strain showed an attenuation of the Cd deleterious effects when co-exposed with PVC, while the SP strain had its survival as reduced as the group solely exposed to Cd (Fig 1).

**Fig 2:**
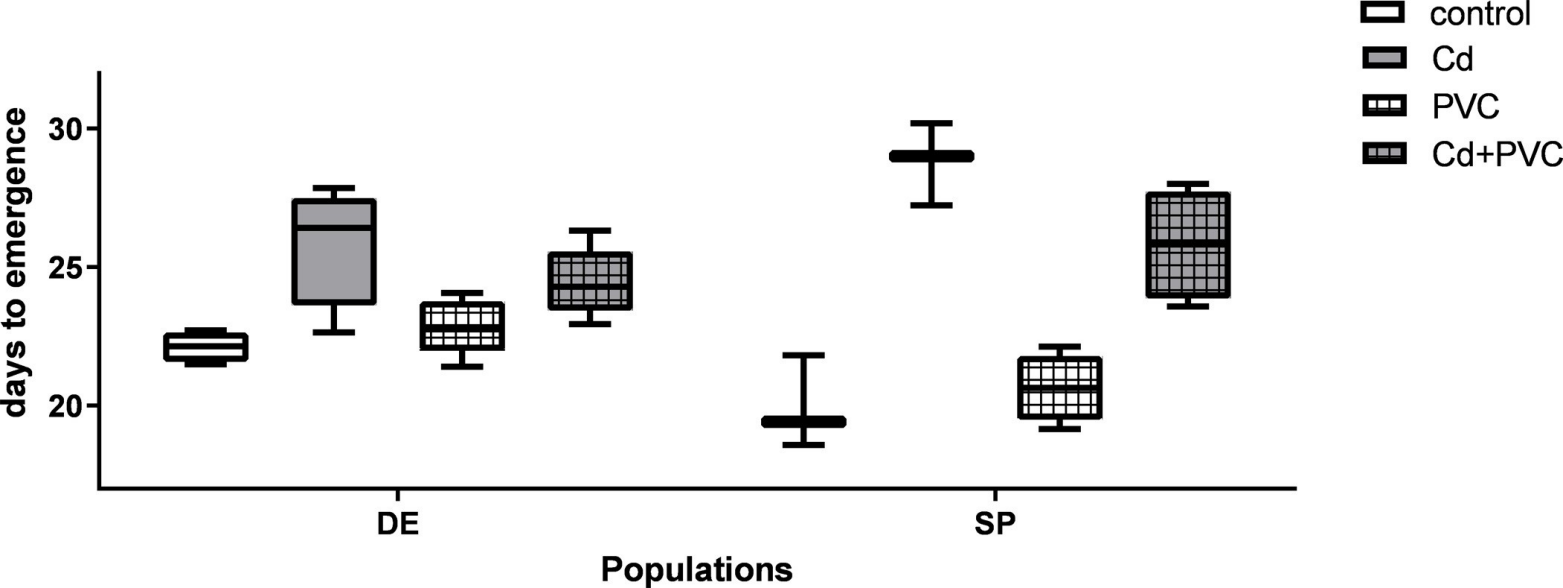
Female median emergence time in *C. riparius* of two different populations originated from Spain (SP) and Germany (DE) after exposure to 50 ug. L^-1^ of Cd (Cd), 1 g.kg^-1^ of PVC MPs (PVC), the mixture of the previous two exposure scenarios (Cd+PVC) and the control group. Data is expressed as mean values ± standard error. Different letters indicate significant differences between groups (p <0.05).

EmT50, the day at which 50% emergence occurred, was completed before 23 days in both controls, meeting the OECD standards. The parameter was affected by the different treatments, but the populations used did not show statistically differences (Fig 2). The Cd treatment had a delayed development of a minimum of 3.5 days respective to the control and PVC exposed groups. The co-exposure of Cd and PVC showed a less pronounced delay, especially in the DE strain, being statistically indistinguishable from the control group. It was not possible to establish a clear difference of developmental speed rates between the two strains due to a high variability of the SP population with a mean value of 19.94±1.7 days against 22.12±0.5 days for the DE population.

**Fig 3:**
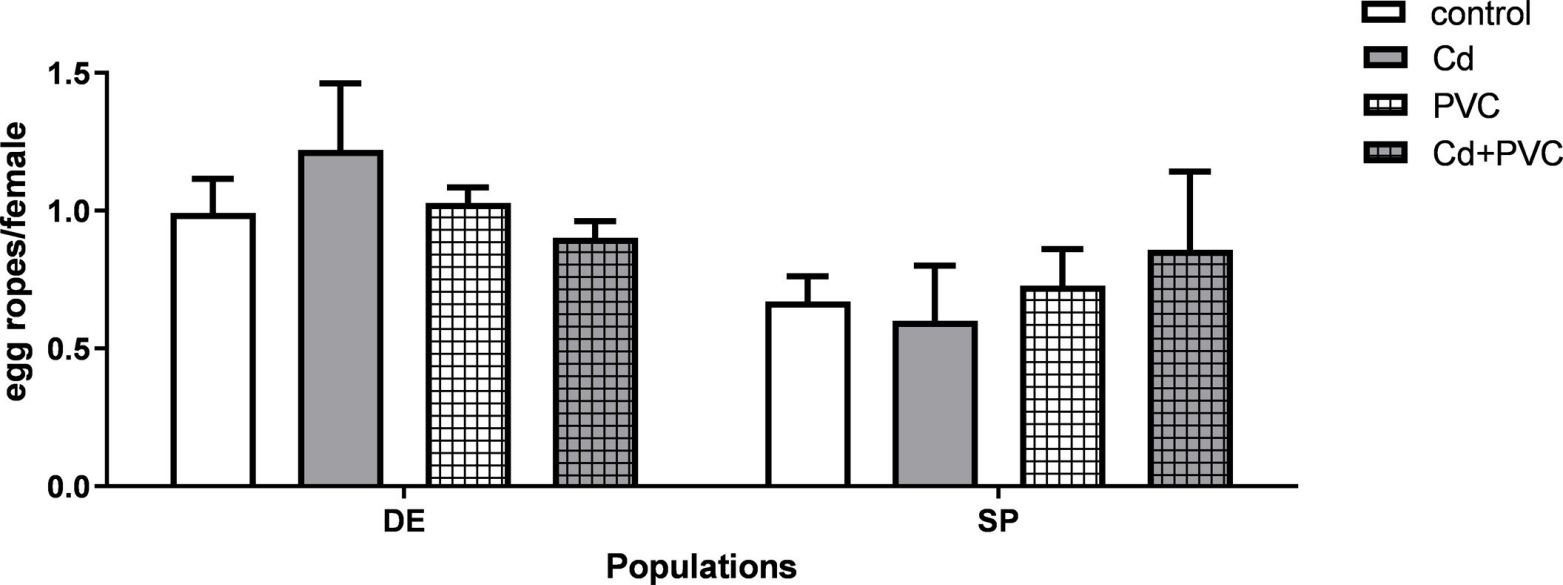
Fertility represented by the number of egg ropes laid per female in *C. riparius* of two different populations originated from Spain (SP) and Germany (DE) after exposure to 50 ug. L-1 of Cd (Cd), 1 g.kg-1 of PVC MPs (PVC), the mixture of the previous two exposure scenarios (Cd+PVC) and the control group. Data is expressed as mean values ± standard error.

Fertility displayed no statistical difference between the different exposure scenarios nor the two laboratory strains (Fig 3).

### 3.2. PGR and linear mixed model

**Fig 4:**
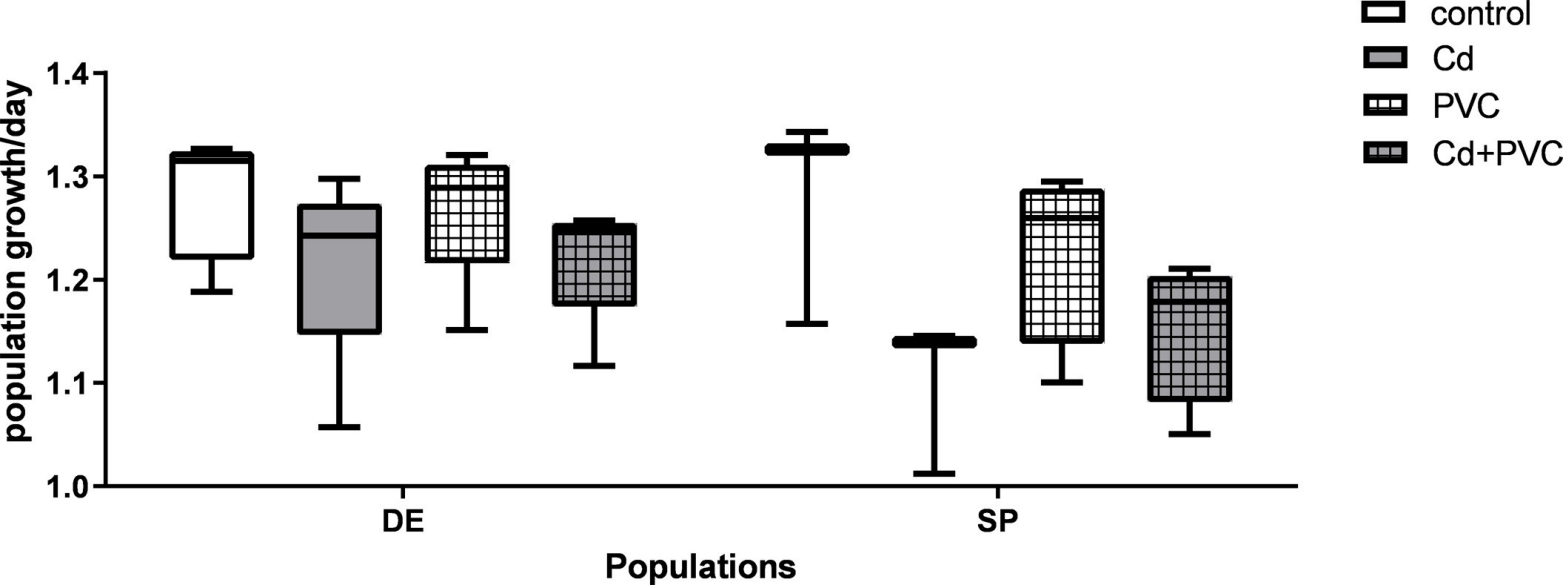
Fertility represented by the number of egg ropes laid per female in *C. riparius* of two different populations originated from Spain (SP) and Germany (DE) after exposure to 50 ug. L-1 of Cd (Cd), 1 g.kg-1 of PVC MPs (PVC), the mixture of the previous two exposure scenarios (Cd+PVC) and the control group. Data is expressed as mean values ± standard error.

Although the ANOVA detected a trend of difference between the different exposure scenarios, a closer comparison with the post-hoc test showed that the PGR variation between treatments or populations were not statistically relevant (Fig 4 and S1 and S2 Tables in S1 File). However, the application of a linear mixed model (Table 1), showed that there is significant interaction between exposure to the treatments and the population used in the tests for the survival and EmT50 parameters, meaning that the use of different strains influenced the outcome of the measured endpoints.

**Table 1:** Summary of the linear mixed model executed for the survival represented as total emergence, EmT50 and fertility.

	Emergence			Female EmT50			Fertility		
	Chisq	*df*	pr(>Chisq)	Chisq	*df*	pr(>Chisq)	Chisq	*df*	pr(>Chisq)
**treat**	53.944	3	1.154e-11 ***	77.029	3	2.2e-16 ***	3.290	3	0.0348
**pop**	16.323	1	5.342e-05 ***	0.001	1	0.976	3.760	1	0.052
**treat:pop**	11.607	3	0.00886 **	18.365	3	0.00036 ***	3.847	3	0.278

Treatment (treat), population (pop) as fixed factor and replicates (replicate per treatments) as random factor. The significance level of the p‐value is displayed as asterisks (***p<0.001, **p<0.01).

## 4. Discussion

Although it was suspected that the choice of experimental populations may have an influence on the results of ecotoxicological tests [17,18], the focus of empirically proving this has long been on comparing natural field populations responses to laboratory strains results [16]. Laboratory populations, regardless of origin or keeping conditions, were always regarded as may be genetically impoverished, but otherwise homogenous [19,42]. The present study aimed thus to investigate if two laboratory strains kept under the same environmental conditions in the same laboratory for more than five years, differing only in their geographical origin and evolutionary history, would show idiosyncratic life-cycle responses to the model pollutants Cd, PVC MPs and a co-exposure with both.

Both strains conformed to classical Cd exposure expectations found in literature by having a lower survival rate and prolonged developmental time even when exposed to low, sublethal concentrations [11,15,43,44]. These known effects might be related to the endocrine disruptor activity of Cd and the promotion of oxidative stress [35,45,46]. Lower fertility, a common outcome of Cd exposure, was not detected in either of the strains. This was in accordance with [19] where Nowak et al. described that different laboratory strains did not differ significantly in the number of egg ropes laid.

Regarding PVC MPs single exposure, neither of the strains showed differences in any end point to the control group. This result was also in accordance with several studies that show that environmentally relevant MPs concentrations are not yet likely to affect measurable life-cycle traits like survival, developmental time, fertility and, consequently, the PGR [9,47–50]. However, even if both DE and SP populations presented similar results when exposed to the single contaminants Cd and PVC MPs, respectively, the co-exposure yielded conflicting results. The Spanish population showed similar results to the Cd-exposure only treatment, whereas the same co-exposure in the DE strain showed no significant adverse effects at all, i.e. it was very similar to the control treatment. It seemed that the co-exposure attenuated the deleterious effects of Cd on the DE strain only. In other words, the two contaminants showed an antagonistic response in the German population, while in the SP strain, the PVC MP co-exposure with Cd showed no interaction at all. In the literature, reports on attenuation effects under co-exposure with MPs are not rare. They vary with the organism evaluated, the type of MP and co-contaminant used in the study and the respective concentrations [4,27,28,51]. Here, we present evidence that such results may not be generally applicable. We rather show that the particular evolutionary history of the study population can decisively influence the outcome of ecotoxicological tests, in this case the sub-lethal toxicity of mixtures.

In the present case, the ultimate reasons for their differential reaction to the same treatment may lie in the differential adaptation to their respective environments of origins and/or differential drift- and adaptation trajectories in the laboratory. Their differential adaptation to climate and temperature was already mentioned [33], but additional factors differentiating the places of origin might have played a role. It is as well possible that, due to the slightly different sampling and restocking history, the two laboratory populations differed in their genetic variability, which might have influenced the results [19]. Previous investigations of *C. riparius* life cycle showed that some populations that occupied more ephemeral water bodies and are, therefore, used to less food availability, have differential food intake and growth rates [52]. On this matter, the interaction between exposure scenarios and population in survival and EmT50 shown in the linear mixed model could point in this direction. Moreover, it would be possible that due to divergent growth rates, the different strains have distinct food intake needs [53]. Thus, since MPs can differentially adsorb and accumulate Cd [28,54], a strain that is more actively dwelling and digging the sediment after food, might ingest more MPs adsorbed with Cd and not beneficiate from any antagonistic effects resulting from this phenomenon. Additionally, another reason for this difference between populations might reside in the differential tolerance of the populations to the single contaminants. Even if the life-cycle responses to the single contaminants were similar, there might be distinct molecular perturbations in the cellular level [9,10]. Indeed, in zebrafish from the same lineage the co-exposure of Cd and MPs showed antagonistic toxicity with low MPs concentration, but synergistic toxicity with high MPs concentration [28].

Not only different origins, but also differential adaptation to the laboratory conditions could have resulted in strain-specific sensitivity. Adaptation to the same (laboratory) conditions have been shown to take differential evolutionary trajectories, even when starting from an identical initial gene-pool [35,55,56].

## 5. Conclusions

Our results add another layer of complexity to ecotoxicological studies by indicating that not only differential evolutionary trajectories of wild populations but also of long-term laboratory strains might play a major role in the outcome of an assessment.

Different laboratory strains, independently from being cultivated in the same environmental conditions and similar period of laboratory existence, might behave differently when challenged by an identical exposure to contaminants. Such an idiosyncratic behaviour of different populations in ecotoxicological assessments is not a bug but an inherent, unavoidable feature of organismic evolution. This calls for independent replications of ecotoxicological assessments and, wherever this is not possible, to take the slightest hint of a detrimental effect seriously–the outcome might be much worse with another experimental population.

## Supporting information

S1 File (DOCX)
