# Supplementary material for: Unveiling population-specific outcomes: Examining life cycle traits of different strains of Chironomus riparius exposed to microplastics and cadmium questions generality of ecotoxicological results
Source: PLoS One. 2024 Jul 10;19(7):e0304739. doi: 10.1371/journal.pone.0304739 (PMC11236181; doi:10.1371/journal.pone.0304739)
Supplement: S1 File — (DOCX) [file pone.0304739.s001.docx]

**SUPPLEMENTARY INFORMATION**

Table 1: ANOVA associated degrees of freedom, F values and p-values for each parameter analyzed.

| **parameter** | **test** | **Factor** | **df** | **test statistic value** | **p-value** |
| --- | --- | --- | --- | --- | --- |
| Survival | Kruskal-Wallis | population | 1 | 3.2126 | 0.07307 |
|  | Kruskal-Wallis | treatment | 3 | 16.946 | 0.0007251 *** |
| EmT50 | Kruskal-Wallis | population | 1 | 0.19107 | 0.662 |
|  | ANOVA | treatment | 3 | 17.19 | 1.32e-06 *** |
| Fertility | ANOVA | population | 1 | 4.025 | 0.0536 |
|  | ANOVA | treatment | 3 | 1.098 | 0.366 |
| PGR | ANOVA | population | 1 | 3.537 | 0.0695 |
|  | ANOVA | treatment | 3 | 3.082 | 0.0428 * |

Asterisks indicate significant differences (***p<0.001; *p<0.05).

Table 2: p-values of the Tuckey’s post hoc test for each comparison between the three treatments.

|  | **Survival** | **EmT50** | **Fertility** | **PGR** |
| --- | --- | --- | --- | --- |
| control-Cd | 0.0017* | 0.0000083*** | 0.4575020 | 0.0646127 |
| Cd+PVC-Cd | 0.5409 | 0.1901814 | 0.4484518 | 0.9629485 |
| PVC-Cd | 0.0568 | 0.0000234*** | 0.4680851 | 0.2127245 |
| Cd+PVC-control | 0.0084** | 0.0012242** | 0.9998235 | 0.1431154 |
| PVC-control | 0.3431 | 0.8789578 | 0.9995000 | 0.8747754 |
| PVC-Cd+PVC | 0.1735 | 0.0046233** | 0.9999844 | 0.4147988 |

Asterisks indicate significant difference between the compared groups (***p<0.001; **p<0.01; p<0.05).
